# Paraquat Toxicogenetics: Strain-Related Reduction of Tyrosine Hydroxylase Staining in *Substantia Nigra* in Mice

**DOI:** 10.3389/ftox.2021.722518

**Published:** 2021-11-11

**Authors:** Carolina Torres-Rojas, Wenyuan Zhao, Daming Zhuang, James P. O’Callaghan, Lu Lu, Megan K. Mulligan, Robert W. Williams, Byron C. Jones

**Affiliations:** ^1^ Department of Genetics, Genomics and Informatics, The University of Tennessee Health Science Center, Memphis, TN, United States; ^2^ Health Effects Laboratory Division, Centers for Disease Control and Prevention-NIOSH, Morgantown, WV, United States

**Keywords:** tyrosine hydroxylase, sporadic Parkinson’s disease, stereology, BXD mice, forward genetic analysis

## Abstract

Paraquat (PQ) is a putative risk factor for the development of sporadic Parkinson’s disease. To model a possible genetic basis for individual differences in susceptibility to exposure to PQ, we recently examined the effects of paraquat on tyrosine hydroxylase (TH)-containing neurons in the substantia nigra pars compacta (SNc) of six members of the BXD family of mice (*n* = 2–6 per strain). We injected males with 5 mg/kg paraquat weekly three times. The density of TH+ neurons counted by immunocytochemistry at 200x in eight or more sections through the SNc is reduced in five of the six strains relative to control (*N* = 4 ± 2 mice per strain). TH+ loss ranged from 0 to 20% with an SEM of 1%. The heritability was estimated using standard ANOVA and jackknife resampling and is 0.37 ± 0.05 in untreated animals and 0.47 ± 0.04 in treated animals. These results demonstrate genetic modulation and GxE variation in susceptibility to PQ exposure and the loss of TH staining in the substantia nigra.

## Introduction

Parkinson’s disease (PD) is a neurodegenerative disorder resulting in progressive motor impairment resulting from selective loss of nigrostriatal dopamine neurons. There are two types of PD, one of which is considered familial, while the other is considered sporadic. Sporadic PD is considered to be caused by some sort of environmental exposure (toxicants) combined with genetic susceptibility (Goldman et al., 2012). The motor symptoms of PD do not start to show until approximately 70–90% of the substantia nigra neurons are lost. Of interest for our research, exposure to environmental toxicants, such as paraquat (PQ), has been associated with late-onset PD or sPD (Ritz et al., 2009). PQ is an herbicide that is widely used for agricultural purposes in the United States and in many other countries. The cases of genetically linked PD are rare compared to sPD (Vila and Przedborski, 2004). Both types of PD share the common manifestation of motor impairment with the underlying accumulation of Lewy bodies and selective loss of nigrostriatal dopamine neurons as main pathophysiological features. The nigrostriatal dopaminergic system plays an essential role in motor control and cognitive function (Klein et al., 2019). Depending on genetic constitution, chronic exposure to a low dose of PQ may present increased risk for developing sPD (Jiménez-Jiménez et al., 1992). Thus, we need a better understanding of the mechanism of toxicity of PQ relative to sPD.

Brain iron accumulation during aging is another putative risk factor for sPD. In the previous work, we showed that PQ disrupts iron homeostasis in the ventral midbrain (containing the SNc) but not the dorsal striatum, by increasing the iron content by about 20% (Yin et al., 2011). Accordingly, we are interested in evaluating the immediate status of these neurons upon initial PQ exposure. PQ toxicity can be influenced by individual genetic makeup. For example, Goldman et al. (2012) showed that PQ-related sPD risk is exacerbated in individuals carrying the null mutant allele of glutathione S-transferase theta isoenzyme. Research on mechanisms that govern neurotoxicity is not always feasible in humans for ethical reasons. Accordingly, we must rely on genetic reference populations of animals, especially mice and rats. This requires exploiting genetically defined animal models that simulate human populations. For example, the BXD family descends from crosses between C57BL/6J (B6) and DBA/2J (D2). This family has great potential for mapping precision due to the high recombination density (Peirce et al., 2004). Presently, the BXD panel contains 140 unique RI strains (Ashbrook et al., 2021), each having been genotyped using high-density SNP arrays, and as such, they are known to segregate for over 5 million common variants and about 12,000 missense mutations, some of which have the potential to affect phenotypes at molecular, cellular, and behavioral levels with relevance to humans (Wang et al., 2016).

Our systems’ genetics approach in *in vivo* experiments leads to a heuristic model of elucidating pathway and biological changes associated with PQ exposure. We previously reported the effects of PQ on regulation of iron, copper, and zinc in the ventral midbrain (VMB), distribution of PQ to the cerebellum, and proinflammatory cytokine gene expression in the cerebellum in 40 BXD strains (Torres-Rojas et al., 2020). Since PD affects the dopaminergic system and tyrosine hydroxylase (TH) is visualized in cell bodies, axons, and terminals in the dopaminergic system (Pickel et al., 1975), here we report the effects of PQ on TH-positive (TH+) neurons in the SNc in BXD mouse strains.

## Materials and methods

### Animals

Male mice from six BXD strains (4 ± 2 mice per strain), obtained from the University of Tennessee Health Science Center, were used in this experiment. The mice were maintained under a constant light–dark cycle (06:00–18:00, on–off rotation), the ambient temperature was 21 ± 2 °C, and the humidity was 35%. The animals were fed with a standard diet, Envigo diet 7912. They also received tap water *ad libitum*. At 6 months of age, the mice were treated with 5 mg/kg paraquat dichloride trihydrate i.p. (PQ, product number 36541, Sigma Chemicals, St. Louis, MO); the solutions were made freshly daily in saline and administered once weekly for 3 weeks. The animals were euthanized the day following the third injection. This PQ dose was sufficient to see accumulation of paraquat in the brain in this mouse model (Torres-Rojas et al., 2020). Control mice were fed the same diet and injected (i.p.) with saline. All procedures were approved by the UTHSC Animal Care and Use Committee.

### Immunohistochemistry

Immediately after sacrifice, mice were perfused with heparinized saline (0.15 M, about 15 ml) followed by fresh ice-cold 4% paraformaldehyde (in 0.1 M phosphate buffer (PB), pH 7.4, about 100 ml). Brains were removed and postfixed in the same solution at 4°C for 24 h and then transferred to increasing concentrations of sucrose (10, 20, and 30%, in 0.1 M PB, pH 7.4) for 24 h each for cryoprotection. Brains were then frozen in isopentane cooled by dry ice and stored at −80°C until sectioning.

The frozen fixed brains were cut using a cryostat (Leica CM-3050-S, chamber temperature −20°C) into 40 μm serial coronal sections containing the entire substantia nigra. Staining was performed by washing three times in PB (0.1 M, pH 7.4). Non-specific binding was blocked by immersion in 1% bovine serum albumin in 0.1 M PB with 0.3% Triton X-100 at room temperature for 1 h. Next, 72 h incubation was performed at 4°C with primary antibody for tyrosine hydroxylase (TH) and anti-neuronal nuclei (NeuN, Fox-3, RBFOX3) which is a nuclear protein expressed in most post-mitotic neurons of the central and peripheral nervous systems. The sections were washed with 0.1 M PB with 0.3% Triton X-10 three times and then incubated with corresponding secondary conjugated antibody for 24 h (detailed information in Table 1). DAPI in anti-fade mounting solution was used as a counterstain for nuclei.

**TABLE 1:** List of antibodies and conditions for the immunohistochemical assay.

First antibody		
Antigen	**NeuN**	**TH**
Host	Mouse	Rabbit
Source	Cell Signaling NeuN (E4M5P) Mouse mAb	Anti-Tyrosine Hydroxylase, Sigma-Aldrich
Catalog number/amount	94,403/100 μl	Ab152/100 μg
Dilution	Suggested 1:500	1:1,000
Second antibody		
Target	Mouse	Rabbit
Host	Goat	Goat
Color	Pink	Red
Source	Cell Signaling/Anti-Mouse IgG (H + L), F(ab’)2 Fragment (Alexa Fluor® 647 conjugate)	Rabbit IgG H&L (Alexa Fluor® 555) preadsorbed
Catalog number/amount	4,410/250 μl	ab150086/500 μg
Dilution	1:1,000	1:1,000
Mounting solution with stain nucleus	ProLong® Gold Antifade Reagent with DAPI	
Color	Blue	
Source	Cell Signaling	
Catalog number/amount	8961S/10 ml	

### Stereology

Images of fluorescence staining for TH+ neurons, neurons, and nuclei in the SNc were captured by using the tile scanning application using a Zeiss LSM 710 confocal microscope system (Berlin, Germany) at 20x. Apparent increase in magnification was accomplished by zoom in Zen 3.3 software. In total, eight or more images were subjected to cell counting for each side of the SNc of the mouse brain. The area corresponding to the SNc was delimited using the Freehand Selection Tool in ImageJ. This area was split into three channels, and TH+ neurons were manually counted on the image corresponding to the red channel using the Point Tool in ImageJ. We verified that TH+ neurons also stained for NeuN and DAPI simultaneously, therefore assuring the counting of TH+ neurons only. Total neurons and nuclei were counted automatically in ImageJ after preparing images with functions subtract background, threshold, and analyze particles, and manual counting was done when needed. We used Zen 3.3 software for image adjusting and amplification. The counting was done blinded by two independent researchers. The interobserver reliability estimate using the Pearson product-moment correlation coefficient was *r = 0.88*, *p <0 .05*.

### Statistical Analysis

All phenotypes were evaluated by analysis of variance (ANOVA) for a two between-subjects variable (strain, PQ) experiment. We report means and standard errors of the mean by strain and dose. Main effects and interactions were considered statistically significant at *α* = 0.05.

## Results

### Paraquat Effects in Neurons of the Substantia Nigra Pars Compacta of BXD Mice

Even though exposures occur in the context of several risk factors, and the interaction between different chemicals and risk factors likely produces different outcomes, we are interested in uncovering the effect of paraquat on SNc neurons in a genetic reference population of mice. For this, we treated the mice with 5 mg/kg of paraquat with weekly i.p. injections for 3 weeks. In our previous study, we demonstrated that this dose showed the maximum variability in the BXD family (Torres-Rojas et al., 2020). We evaluated whether paraquat had selectivity for TH staining by measuring not only TH+ neurons but also post-mitotic neurons (NeuN) and nuclei (DAPI).

**FIGURE 1:**
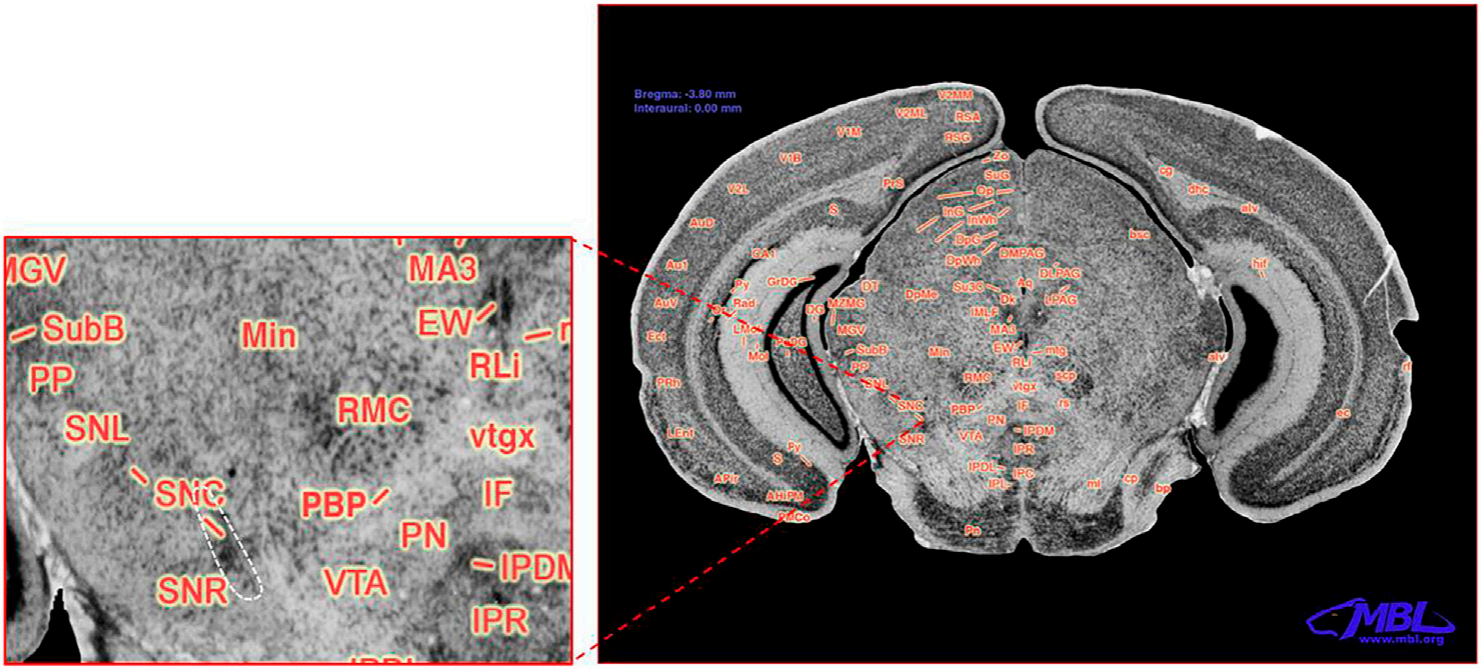
Coronal section of the C57BL/6J mouse brain. Modified with permission from ^©^1999 RW Williams, design by AG Williams, atlas by T Capra, at http://www.mbl.org/atlas170/large_label/24.jpg.

**FIGURE 2:**
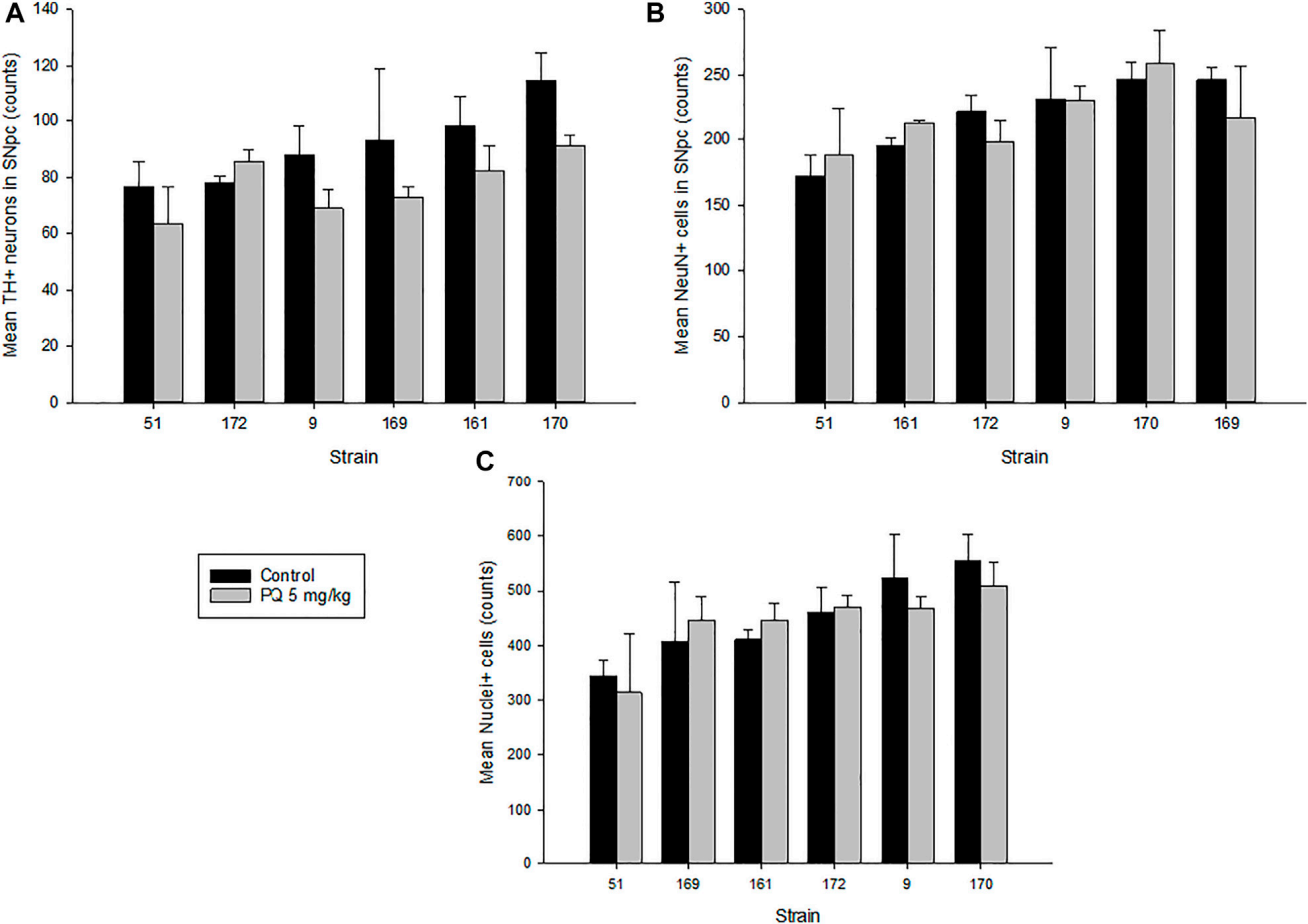
Quantitative results of SNc stereological analysis. **(A)** Tyrosine hydroxylase–positive (TH+) neurons in the SNc of mice treated with 5 mg/kg of paraquat and control mice. Controls showed an average of 91(22) counts in TH+ neurons versus 79(14) counts in treated mice. **(B)** Neuronal staining–positive cells (NeuN+) in the SNc of treated mice versus control. **(C)** Nuclei staining–positive cells (Nuclei+) in the SNc of treated versus control mice. Values are expressed as mean ± SEM and mean (SD) of cell counts. Bars are grouped in ascending order by controls.

The brain images for cell counting are based on the schematic of the area containing the SNc shown in Figure 1, and the white-dashed area in the zoom panel represents the *substantia nigra pars compacta* marked as SNc (Williams et al., 1999). The analysis of variance revealed significant main effects of strain and treatment but not their interaction on TH+ neurons (F_5,42_ = 3.589, *p* < 0.012; F_1,42_ = 9.375, *p* < 0.01; F_5,42_ = 1.230, *p* < 0.32, respectively). Figure 2A and Table 2 present stereological cell counts of SNc TH+ neurons in PQ-injected mice, revealing loss of TH+ neurons in most strains tested. This TH+ loss ranged from 0 to 20% with an SEM of 1%. There were no significant effects of strain, treatment, or their interaction on neuron counts (Figure 2B) (F < 1 for all). There was a main effect of strain on nuclei counts (Figure 2C) (F_5,42_ = 4.38, *p* < 0.005) but not of treatment or strain × treatment interaction (F < 1 for both). We obtained low correlation between TH+ neurons and NeuN, r = 0.321, *p* < 0.04, and a higher correlation between NeuN and nuclei, r = 0.62, *p* < 0.001. TH+ loss ranged from 0 to 20% with an SEM of 1%. The heritability was estimated using standard ANOVA and jackknife resampling and is 0.37 ± 0.05 in untreated animals and 0.47 ± 0.04 in treated animals. Jackknife resampling is used to evaluate the variance and therefore the precision for an estimate when the sample is small. Heritability results indicated there is a genetic modulation in susceptibility to PQ effects on TH+ neuronal staining.

**TABLE 2:** Descriptive statistics for the major phenotypes.

Strain/phenotype	BXD9	BXD51	BXD161	BXD169	BXD170	BXD172
TH_CONTROL	87.9067	76.785	98.308	93.15	114.8067	78.195
SE	10.48896	8.79791	10.85887	25.65	9.7083	2.33781
N	3	4	5	2	3	4
TH_PQ5	68.9775	63.45	82.3667	72.76	91.255	85.715
SE	6.76838	13.02	8.91364	3.65551	3.69761	4.2333
N	4	2	3	4	4	6
NEUN_CONTROL	231.7300	172.5200	195.3625	246.7500	246.2833	221.1800
SE	38.77711	15.36386	6.361,845	8.75	13.96373	13.01852
N	3	4	4	2	3	4
NEUN_PQ5	230.745	187.8	212.78	216.52	258.6925	198.585
SE	10.49685	35.8	1.700,863	40.28408	25.41679	15.79633
N	4	2	3	4	4	6
NUCLEI_CONTROL	523.04	345.08	410.885	407.96	554.5567	461.2125
SE	79.07568	26.99431	17.59993	107.96	48.45653	45.82473
N	3	4	4	2	3	4
NUCLEI_PQ5	468.625	313.965	446.86	446.8475	508.535	469.48
SE	21.39394	108.105	30.82871	41.54778	43.99676	21.82583
N	4	2	3	4	4	6

TH, tyrosine hydroxylase; NEUN, anti-neuronal nuclei; NUCLEI, count of cell nuclei stained with DAPI. Data are presented as the mean (counts), standard error (SE), and number of mice per group and strain (N).

**FIGURE 3:**
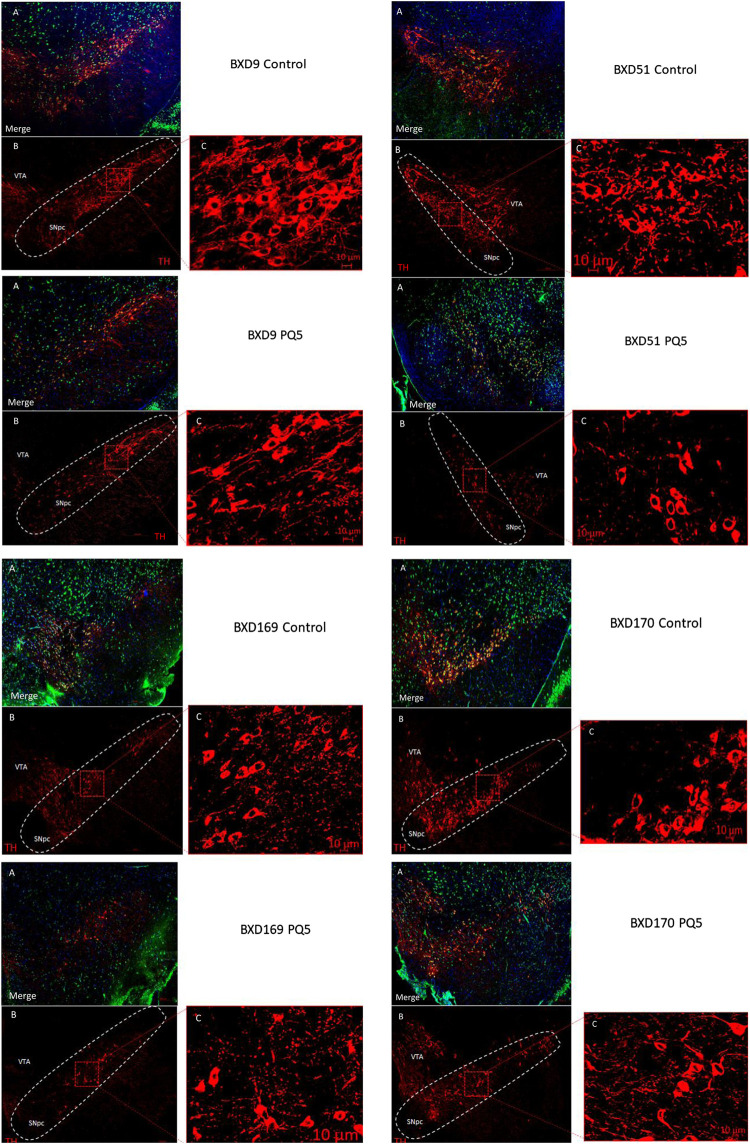
Stereological photomicrographs of cell counting in the SNc. Representative images of 40 μm SNc sections from a control mouse **(upper panel)** and PQ-treated mouse **(lower panel)**. **(A)** Merged image for TH+ (red cells), NeuN (green cells), and DAPI (blue cells). **(B)** Immunostaining for TH. **(C)** Zoom of TH+ neurons in the highlighted area of **(B)** by Zen program. The scale bar is 200 μm. In the TH image, the adjacent area corresponds to the ventral tegmental area (VTA), and the delimited area represents the SNc (or SNpc).

Representative immunofluorescence analyses (Figure 3) revealed that PQ treatment reduced TH staining (lower panel) compared to control (upper panel) in most BXD strains, the SNc area was delimited for cell counting, and the ventral tegmental area (VTA) was left outside of the counting. The red TH image shows cells that stained positive for tyrosine hydroxylase, the merged image shows three color images merged for TH, neuronal, and nuclear staining, and zoom panels show the area within the SNc delimited with the dashed red square. Overall, these data suggest that paraquat has selectivity for TH staining in the SNc and may not necessarily affect in the short term other populations of post-mitotic neurons or cells that show positive staining for NeuN and DAPI.

## Discussion

The ultimate goal of neurotoxicogenetics is the understanding of molecular and biochemical mechanisms underlying individual differences in susceptibility to effects of toxicants. Previously, we showed that the parental strains for the BXD panel evinced differential sensitivity to PQ effects on TH+ neuron staining with B6 showing greater loss than D2 mice (Yin et al., 2011). By adding more strains here, we strengthen the proof of principle of genetic-based individual differences in the neurotoxic effects of PQ. Our observation of decreased TH+ neuron staining and absent loss of neurons or other cells in the SNc raises the issue of whether PQ actually destroys TH+ neurons in this area. Future work is needed to ascertain whether the loss of TH+ neuron staining is permanent or reversible and, if permanent, whether PQ prevents the synthesis of TH but spares the neuron.

No doubt, there are several ways and exposures that underlie sPD, and we have elucidated only one. Whether similar genetic profiles confer differential risk across different exposures is compelling and remains to be examined. TH expression in adult SNc neurons can be easily regulated by several factors including neurotoxicants and endogenous proteins that are potentially essential for neuron survival. We do not know how long the observed changes in TH staining last after PQ insult and whether they disappear or exert additional effects in the long term. Others have demonstrated that altered neuronal activity changes TH expression, and as a compensatory mechanism, the number of TH+ SNc cells can be inversely proportional to the change in TH expression (Aumann et al., 2011). PD models with 1-methyl-4-phenyl-1,2,3,6-tetrahydropyridine reported decreased TH expression in the SNc with no evident neuron loss after 7 days of post-single low dose exposure (Alam et al., 2017). Therefore, it would be interesting to measure whether the variable changes in TH staining due to PQ exposure can last more than few days and what permanent consequences this can imply to the dopaminergic system.

As concerns mechanisms by which PQ can exert TH toxicity, one possible means is by PQ disrupting iron homeostasis in the SNc. Previously, we have shown that PQ does disrupt iron homeostasis in the SNc (Yin et al., 2011; Torres-Rojas et al., 2020). We have also shown that MPTP/MPP+ disrupts iron homeostasis in a mouse strain that shows greater sensitivity to MPTP toxicity (Jones et al., 2013).

As a matter of interest in the present findings, there are some limitations. First, we were limited in the number of strains, and this was partly because cell counting is extremely time-consuming. Additionally, we had samples from males only, and this was because of budgetary concerns. Future work will involve at least 30 strains and both sexes. Thirty strains are minimal for identification of candidate genes that underlie individual differences in sensitivity, and the inclusion of both sexes is necessary because in some toxicological studies, we observe similarities and important sex differences (Alam et al., 2016). Finally, it is important to note that while we assert strain differences in PQ effects on TH staining, we did not observe a significant strain × treatment interaction by ANOVA. This is not surprising as ANOVA tends to be underpowered for detecting gene × environment interactions (Wahlsten, 1990). Nevertheless, it is evident from our previous work that PQ can get to the mouse brain, and based on those results, here we examined the PQ variable effect on a feature that is relevant to PD *via* immunofluorescence. This work expands stereological observations in strains of the BXD family. The notion of genetic-based individual differences in susceptibility to PQ neurotoxicity is supported, and expansion of the work to include more strains and molecular techniques is warranted.

## Conclusion

Here, we provide more evidence that the neurotoxicity of paraquat shows genetic-based individual differences in an animal model with likely translation to humans in support of extant evidence (Goldman et al., 2012). Individual differences in host susceptibility also help explain the apparent disparate results from epidemiological studies (Jones et al., 2014). Finally, these results beg further study to elucidate the actual genes and gene networks that underlie the individual differences in susceptibility, thus pointing to the biochemical pathways and mechanisms of paraquat neurotoxicity.

## Data Availability

The original contributions presented in the study are included in the article/Supplementary Material, and further inquiries can be directed to the corresponding author.
